# Factors Related to Advanced Stage of Cancer Presentation in Botswana

**DOI:** 10.1200/JGO.18.00129

**Published:** 2018-12-11

**Authors:** Chidinma Anakwenze, Rohini Bhatia, William Rate, Lame Bakwenabatsile, Kebatshabile Ngoni, Sarah Rayne, Preet Dhillon, Mohan Narasimhamurthy, Ari Ho-Foster, Doreen Ramogola-Masire, Surbhi Grover

**Affiliations:** **Chidinma Anakwenze**, MD Anderson Cancer Center, Houston, TX; **Rohini Bhatia**, University of Rochester School of Medicine and Dentistry, Rochester, NY; **William Rate**, Georgetown University School of Medicine, Washington, DC; **Lame Bakwenabatsile**, **Ari Ho-Foster**, and **Surbhi Grover**, Botswana-University of Pennsylvania Partnership, Gaborone, Botswana, and Philadelphia, PA; **Kebatshabile Ngoni**, **Mohan Narasimhamurthy**, and **Doreen Ramogola-Masire**, University of Botswana; **Surbhi Grover**, Princess Marina Hospital, Gaborone, Botswana; **Sarah Rayne**, University of the Witwatersrand, Johannesburg, South Africa; **Preet Dhillon**, Public Health Foundation of India, Gurgaon, Haryana, India; and **Ari Ho-Foster, Doreen Ramogola-Masire** and **Surbhi Grover**, University of Pennsylvania, Philadelphia, PA.

## Abstract

**Purpose:**

Botswana, a country with a high prevalence of HIV, has an increasing incidence of cancer-related mortality in the post–antiretroviral therapy era. Despite universal access to free health care, the majority of Botswana patients with cancer present at advanced stages. This study was designed to explore the factors related to advanced-stage cancer presentation in Botswana.

**Methods:**

Patients attending an oncology clinic between December 2015 and January 2017 at Princess Marina Hospital in Gaborone, Botswana, completed a questionnaire on sociodemographic and clinical factors as well as cancer-related fears, attitudes, beliefs, and stigma. Odds ratios (ORs) were calculated to identify factors significantly associated with advanced stage (stage III and IV) at diagnosis.

**Results:**

Of 214 patients, 18.7% were men and 81.3% were women. The median age at diagnosis was 46 years, with 71.9% of patients older than 40 years. The most commonly represented cancers included cervical (42.3%), breast (16%), and head and neck (15.5%). Cancer stages represented in the study group included 8.4% at stage I, 19.2% at stage II, 24.1% at stage III, 11.9% at stage IV, and 36.4% at an unknown stage. Patients who presented at advanced stages were significantly more likely to not be afraid of having cancer (OR, 3.48; *P* < .05), believe that their family would not care for them if they needed treatment (OR, 6.35; *P* = .05), and believe that they could not afford to develop cancer (OR, 2.73; *P* < .05). The perception that symptoms were less serious was also significantly related to advanced stage (*P* < .05). Patients with non–female-specific cancers were more likely to present in advanced stages (OR, 5.67; *P* < .05).

**Conclusion:**

Future cancer mortality reduction efforts should emphasize cancer symptom awareness and early detection through routine cancer screening, as well as increasing the acceptability of care-seeking, especially among male patients.

## INTRODUCTION

As low- and middle-income countries experience population growth and reduced mortality from communicable diseases, their cancer burdens increase. By 2030, cancer rates will nearly double in some low- and middle-income countries where screening programs are scarce, health systems are poorly equipped, and awareness is limited.^1^ Because of these limitations, patients often present with advanced-stage malignancies, which leads to greater rates of cancer-related deaths.^2^

Botswana, a middle-income country in southern Africa with an HIV prevalence of 21.9% among adults 15 to 49 years of age, has an increasing incidence of cancer-related mortality in the post–antiretroviral therapy era.^3^ With a population of approximately 2 million people, Botswana has 1,600 new patients with cancer per year.^1^ The country's name means land of the Tswana, referring to the dominant ethnic group in Botswana. However, the term Batswana is used generally as a demonym for all citizens of Botswana.^4^

Cancer screening is not common in the public sector of Botswana. Prior studies have reported low rates of mammography screening, likely because mammography is not readily available and thus is not part of routine screening.^5^ Prostate cancer screening is also not routine.^6^ Similarly, colon cancer screening is not commonly performed, partly because the incidence of colon cancer in Botswana is low compared with higher-income countries.^1,7^ However, efforts to bolster cervical cancer screening have been made; for example, See and Treat, a program involving visual inspection after acetic acid application to the cervix, was implemented for HIV-infected women.^8,9^ Additional efforts to curb the incidence of cervical cancer have also included a government-funded comprehensive human papillomavirus vaccination plan.^10^ Despite universal access to government-funded health care, the majority of Batswana patients with cancer, half of whom are infected with HIV, present at advanced stages.^11^ Although antiretroviral therapy coverage has reached 83% in Botswana, and median CD4 counts in previously published literature from the antiretroviral therapy era demonstrate a well-managed HIV population, HIV-infected Batswana individuals remain three to five times more likely to develop cancer than age-matched HIV-negative controls.^3,8,12,13^ Surprisingly, even HIV-infected patients with cancer with regular longitudinal contact with the health care system do not have faster linkages into cancer care.^11^ Median time from cancer symptom onset to treatment initiation in Botswana was reported to be 13 months, compared with 3 months in more developed settings.^11,14,15^

Prior studies suggest that delays in oncologic treatment may be related to distance from the hospital, health insurance status, quality of health care systems, use of traditional healers, financial opportunity costs, limited cancer awareness, and cancer stigma or fear.^16-18^ A prospective study conducted in Botswana suggested that compared with patients with early-stage disease, patients with rapidly progressing symptoms and advanced disease entered into specialized oncology treatment earlier after initial symptom recognition.^11^ Perhaps additional improvements can be made through earlier symptom recognition at the individual or clinic level. However, additional efforts are needed to understand factors associated with advanced-stage presentation.

Given the existing literature, we suspect that the reason for advanced presentation is multifactorial. Therefore, we sought to describe sociodemographic and clinical factors, as well as the knowledge, attitudes, and beliefs associated with advanced stage at diagnosis in Botswana. We believe that understanding factors associated with advanced-stage presentation is crucial to facilitating earlier cancer detection and intervention, thus reducing cancer-related mortality in Botswana.

## METHODS

### Procedure

This was a cross-sectional study conducted from December 2015 to January 2017 at the Princess Marina Hospital in Gaborone, Botswana. This hospital provides oncology care for the majority of patients in southern Botswana. The study population consisted of a convenience sample of 214 newly diagnosed patients who were at least 18 years old and presented to Princess Marina Hospital for initial cancer treatment with a pathologically confirmed diagnosis of cancer.

Patients were approached by a member of the research team and asked whether they would like to participate in a study assessing delays in cancer care. The questionnaire was administered in Setswana and English, and research assistants administered the survey to patients who were illiterate in the study language. The study protocol was approved by the Institutional Review Board at the University of Pennsylvania and the Health Research Development Committee at the Botswana Ministry of Health. Written informed consent was obtained from each participant before completion of the questionnaire.

### Study Measures

The first section of the questionnaire consisted of sociodemographic questions, including age, sex, relationship status, literacy level, and educational attainment. We also assessed the presence of comorbidities (diabetes, HIV, and tuberculosis), distance to the hospital, symptom severity, and cancer site (Table 1). Additional questions assessed place of residence, languages spoken, employment, economic status, ability to take time off from work, assets (home, land, or livestock ownership), family size, methods of transportation, travel time to the hospital, and religious background (Table 2).

**Table 1:**
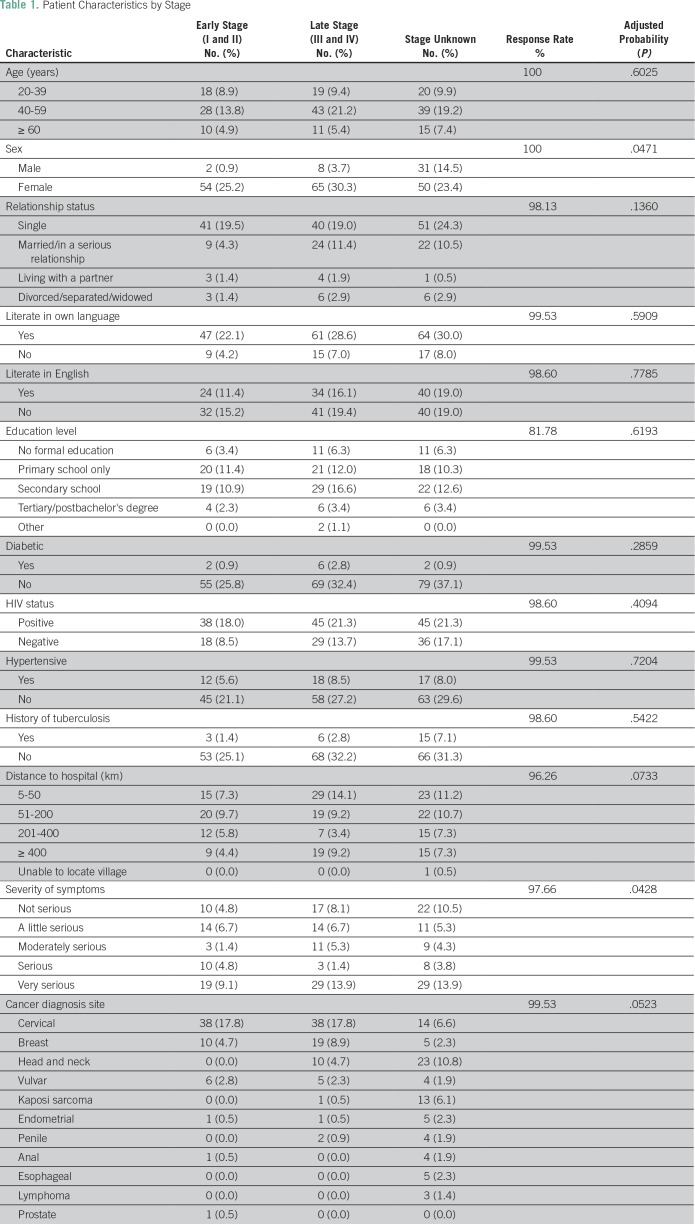
Patient Characteristics by Stage

**Table 2:**
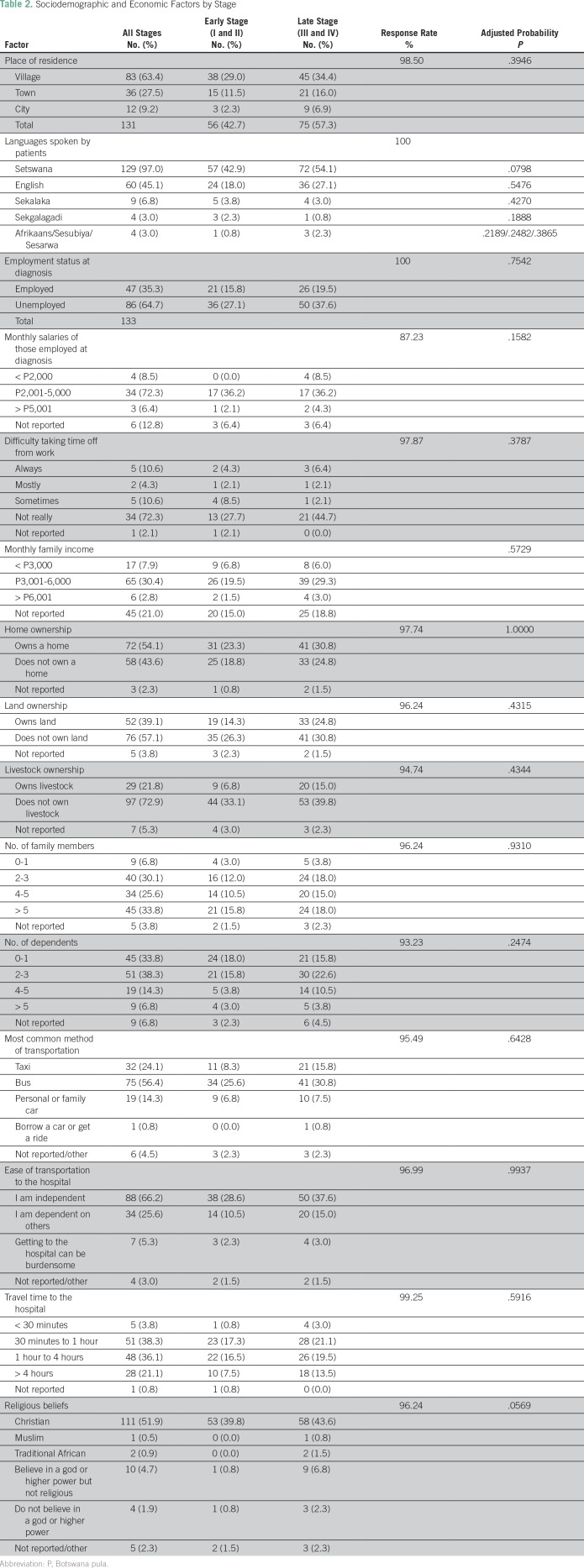
Sociodemographic and Economic Factors by Stage

The final section was adapted from a prior study among patients with breast cancer in South Africa. It assessed cancer-related fears, attitudes, beliefs, and stigma using a four-point summative scale ranging from strongly agree to strongly disagree (Table 3).^19^ For the analysis, patients who agreed or strongly agreed were categorized as agree and patients who disagreed or strongly disagreed were categorized as disagree. The questionnaire was piloted with Batswana women to further refine it and adapt it to Botswanan culture.

**Table 3:**
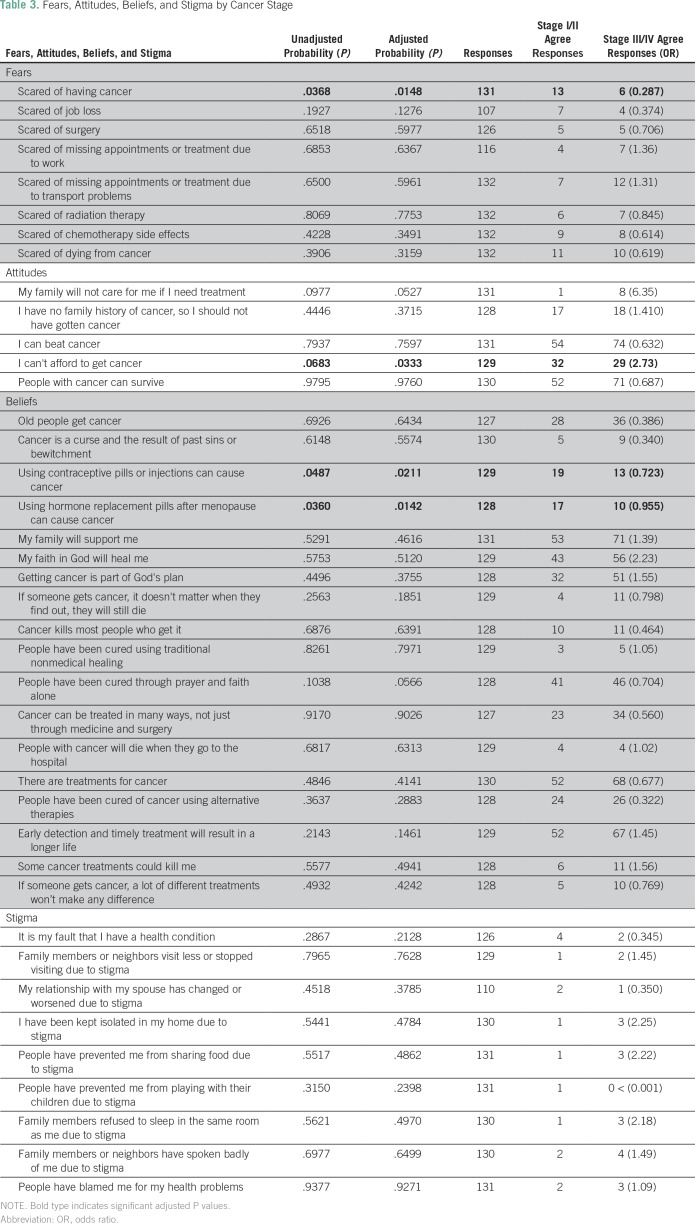
Fears, Attitudes, Beliefs, and Stigma by Cancer Stage

Cancer diagnosis was made using pathologic confirmation. Cancers were staged according to the TNM staging system of the American Joint Commission on Cancer, seventh edition.^20^ Staging evaluation varied by cancer site but often included medical chart review, physical examination, and imaging with chest x-ray and ultrasound. Cancers were categorized as early (stage I and II) or advanced (stage III and IV). However, some patients were characterized as being in the unknown stage if they were not staged before treatment initiation or if imaging modalities were not functional at the time of diagnosis.

### Statistical Analyses

Responses to the questionnaire were collected electronically using REDCap (Research Electronic Data Capture) tools hosted at the University of Pennsylvania.^21^ All statistical analysis was completed using commercially available analytic software (STATA, version 15.0; STATA, College Station, TX).

Nonparametric post hoc one-way analysis of variance between all measured and calculated variables for patients with early-stage disease (stage I and II) and advanced-stage disease (stage III and IV) were analyzed using the Kruskal-Wallis H test, which adjusts for ties between ordinal responses to a question. Unadjusted and adjusted probabilities were reported to indicate the significance of the difference between recorded categorical patient responses in the early- and advanced-stage groups (Table 3). Univariable logistic regression was used to determine directionality of the difference between early- and advanced-stage group responses to the fears, attitudes, beliefs, and stigma portion of the survey, as well as unadjusted odds ratios (ORs) describing the impact of our population’s agreement or disagreement with survey questions on their relative probability of being in the advanced-stage group (Tables 3 and 4).

**Table 4:**
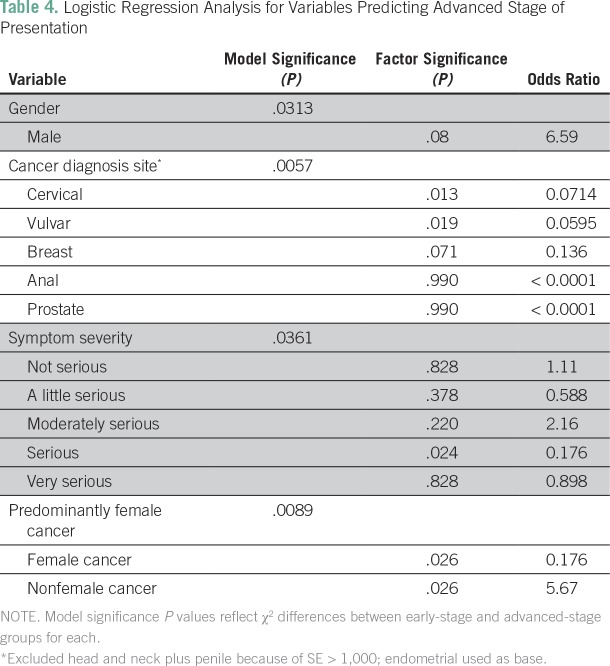
Logistic Regression Analysis for Variables Predicting Advanced Stage of Presentation

A multivariable logistic regression analysis was performed for each variable contained in the surveys to determine the significance of any single item’s ability to predict advanced stage at diagnosis in a post hoc fashion. The significance of both the model and the categorical factors is listed for all items producing a significant predictive model in Table 4, with associated ORs. Any categorical factors with an SE of greater than 1,000 were excluded from the logistic regression performed on a variable.

## RESULTS

### Demographics

A total of 214 patients presenting for specialized cancer care were included in the analysis. Of the 214 patients, 57 (28.2%) were younger than 40 years of age, 40 (18.7%) were male, and 174 (81.3%) were female. Half of the patients (50.3%) reported at least a secondary school level of education, and 55 (26.2%) reported being married or living with a partner. The most commonly represented cancers were cervical (n = 90; 42.3%), breast (n = 34; 16%), head and neck (n = 33; 15.5%), vulvar (n = 15; 7%), and Kaposi sarcoma (n = 14; 6.6%). The remaining 27 malignancies (12.7%) included endometrial, penile, anal, esophageal, lymphoma, and prostate. At least 49% of the patients with cervical cancer, 11% of the patients with breast cancer, 11% of the patients with head and neck cancer, and 9.5% of the patients with vulvar cancer and Kaposi sarcoma were HIV positive. All other cancers had less than 5% of patients who were HIV positive. Cancer stages represented in the study group included 17 (8.4%) at stage I, 39 (19.2%) at stage II, 49 (24.1%) at stage III, 24 (11.9%) at stage IV, and 74 (36.4%) with unknown stage. Other key patient characteristics are listed by stage in Table 1.

### Socioeconomic Factors

Multiple languages were represented in the patient sample, with 129 patients (97%) speaking Setswana and 60 (45.1%) speaking English, normally as a second language. Most patients reported no transportation problems, with only 17 patients (12.8%) with early-stage cancer and 24 patients (18%) with advanced-stage cancer reporting that getting to the hospital was burdensome or that they were dependent on others to get to the hospital. Most of the patients (n = 86; 64.7%) who responded to the employment question were unemployed at the time of diagnosis. Of those who were employed, 34 (72.3%) earned in Botswana pula (P) between P2,001 and P5,000 per month ($209 to $522 in US dollars in June 2016). Other socioeconomic factors are listed by stage in Table 2. No significant relationships were noted between any socioeconomic variable and advanced-stage disease.

### Fears, Attitudes, Beliefs, and Stigma

Patients who were not afraid of having cancer were more likely to present with advanced-stage cancer at diagnosis (OR, 3.48; *P* < .05). Patients who agreed that their family would not care for them if they needed treatment were more likely to have advanced-stage disease (OR, 6.35; *P* = .05). Patients who agreed that they could not afford to develop cancer were more likely to present with advanced disease (OR, 2.73; *P* < .05). Other fears, attitudes, beliefs, and stigmas are listed by stage in Table 3.

### Factors Associated With Advanced Stage in Multivariable Logistic Analysis

Patients with non–female-specific cancers were more likely to present with advanced-stage disease (OR, 5.67; *P* < .05). Female-specific cancers were defined as cancers of the female reproductive organs (cervical, ovarian, uterine, vaginal, vulvar) and breast. There was also a nonsignificant trend toward male patients presenting with advanced-stage cancer (OR, 6.59; *P* = .08; Table 4). Patients with cervical and vulvar cancer were less likely to present with advanced stages (OR, 0.07 and OR, 0.06, respectively; *P* < .05). Patients who reported serious symptom severity at presentation were less likely to have advanced-stage disease (OR, 0.176; *P* < .05).

## DISCUSSION

Half of the study participants at the Princess Marina Hospital presented with advanced-stage cancers. Understanding factors associated with advanced-stage presentation is crucial to facilitating earlier cancer detection and intervention, thus reducing cancer-related mortality. This is particularly important in cancers that are preventable through screening and vaccination, such as cervical cancer, human papillomavirus–related cancers, and breast cancer, which typically present at an advanced stage in Botswana and comprise more than half of the study population when combined.

Our study population is representative of the general population when considering age, sex, socioeconomic status, and cancer type. For example, the median age at diagnosis was 46 years, with 71.9% of patients older than 40 years. This is consistent with Botswana National Cancer Registry data reporting that the median age at cancer diagnosis was 47 years for women and 50 years for men.^22^ Our study also showed that 72.3% of the participants earned between P2,001 and P5,000 per month, which is within the limits of the average monthly salary of Batswana (P4,801) during the study period.^23^ The most commonly represented cancers included cervical (42.3%) and breast (16%). These data are consistent with existing cancer incidence data, which show that cervical and breast cancer are the most commonly diagnosed cancers, as well as the most common causes of cancer death, representing 15.3% and 9.5%, respectively, of all newly diagnosed cancers. However, given the large number of female participants in our study, cervical and breast cancers had higher representation. Patients with Kaposi sarcoma comprised 6.6% of our sample, which is consistent with existing data that show Kaposi sarcoma represents 7.6% of all newly diagnosed cancers.^1^

There is relatively good access to specialized cancer care in Botswana, with government-funded care being accessible to 90% of the population.^8^ Although patients with HIV might have closer linkages to the health care system through Botswana’s robust antiretroviral therapy program, our study did not show an association between HIV status and earlier stage at diagnosis. This is consistent with prior data that suggest that even HIV-infected patients with cancer with regular contact with the health care system do not have faster linkages into cancer care.^11^ This also suggests that there is a need to indiscriminately bolster existing methods for early diagnosis, provision of quality care, and efficient management of limited resources in patients with HIV-positive and HIV-negative disease. It is important to note that as antiretroviral therapy coverage has increased from 7.3% to 82.3% between 2003 and 2008, age-adjusted cancer incidence has decreased in patients with HIV by 8.3% per year. However, with a progressively larger and older HIV population, there is still a high number of incident cancers in the HIV population.^22^

Prior studies have suggested that advanced-stage presentation may be related to difficulty getting to the hospital; however, our study found that difficulty getting to the hospital was not a cause of advanced-stage presentation.^16,18^ This suggests that there may be other prevailing causes in Botswana. For example, gender-related factors predicted increased stage at presentation. A qualitative meta-analysis reported that men often view help-seeking as unmasculine, and women find help-seeking easier, given their greater contacts with health services for themselves and their families.^18^ One study reported that of 5,000 monthly patients attending a clinic in Gaborone, Botswana, 60% were female. Women were seen more frequently than men for reasons such as sexual and reproductive health, pre-employment medical examination, and consultation for various new symptoms.^5^ The discrepancies between male and female stage of presentation may also be related to differences in symptoms between gender-specific cancers (ie, vaginal bleeding may be more disturbing than urinary symptoms in prostate cancer).

In this study, patients who perceived their symptoms to be serious were more likely to present at early stages. This is consistent with prior studies that suggest that severe symptoms, symptoms that interfere with everyday life, or well-recognized symptoms (ie, lump) are potent triggers of early illness recognition and help-seeking.^18,24^ In contrast, patients with vague or nonspecific initial symptoms are known to have delayed illness recognition.

We also found that certain fears, attitudes, and beliefs were predictive of advanced stage at presentation. Patients who were not afraid of having cancer were more likely to present with advanced-stage disease. This suggests that those who present with advanced-stage cancer may not understand the mortality risks associated with a cancer diagnosis or may not be concerned about this within their belief structures. This is a direct contradiction of the other highly prevalent belief that early detection and early treatment results in a longer life. These contradicting beliefs represent a cognitive dissonance that aids in the avoidance of their own mortality, particularly in advanced-stage cancer. Most of the participants also felt that their faith would cure their disease. This belief in faith-based cure has been previously reported in other African countries.^19^ Patients who believed that their family would not care for them or that they could not afford to develop cancer were more likely to present with advanced-stage disease. This is consistent with prior studies that suggest responsibility for the needs of other family members often prevents patients from prioritizing their own health needs.^18^ Often, patients do not seek health care until their symptoms start to affect their ability to work.^25,26^ These findings suggest that future interventions should target increasing cancer symptom awareness and promotion of early care-seeking among primary wage earners in the family.

Most of the patients with early- and advanced-stage disease held appropriate fears, attitudes, and beliefs regarding cancer and its treatment options. They also indiscriminately reported low levels of cancer stigmatization in their homes and communities, which is consistent with prior African studies that report low levels of cancer stigma.^27-29^ This is important to note because stigma has been previously reported as a potential barrier to participation in cancer screening or cancer care-seeking activities.^30^ Although interventions targeted toward reducing stigma might improve the psychosocial well-being, interpersonal relationships, and financial opportunities for patients with cancer and can even increase early health care–seeking behaviors, the lack of stigma experienced in this patient population suggests that an emphasis on stigma reduction may not play an important role in the Botswana population of patients with cancer.^31,32^

This study is not without limitations. The sample size was small, which may affect the reliability of the survey’s results. Individuals with early-stage cancer may not know that they are sick, and thus may not be well represented in the study sample. Furthermore, we were only able to capture patients who present for care. In addition, self-reported fear, attitude, belief, and stigma data might be biased because of social desirability and recall bias after diagnosis. Issues with survey-item nonresponse may also introduce bias; however, the majority of survey items had a greater than 90% response rate. We do not have data on patients who refused to participate in the study. Patient refusal could present as a study limitation; however, it is unclear in which direction this might bias the study. Last, the heterogeneity of the cancer sites makes it difficult to form cancer site-specific conclusions that may reduce advanced-stage cancer presentation in the cancer site of interest. Regardless, these findings are important and provide significant insight into causes of advanced-stage cancer presentation in Botswana. 

This study examined the sociodemographic and clinical factors, as well as the knowledge, attitudes, and beliefs, associated with delayed stage at diagnosis in Botswana. Patients who presented at advanced stages were more likely to not be afraid of having cancer, believe that their family would not care for them if they needed treatment, and believe that they could not afford to develop cancer. Advanced stage at presentation was found to be associated with non–female-specific cancers and the perception that symptoms were less serious. Future cancer mortality reduction efforts should emphasize cancer symptom awareness and early detection through routine cancer screening, as well as increasing the acceptability of care-seeking through education about cancer outcomes if detected early, especially among male patients.
